# Crystal structure of 2′-[(2′,4′-di­fluoro­biphenyl-4-yl)carbon­yl]-1′-phenyl-1′,2′,5′,6′,7′,7a’-hexa­hydro­spiro­[indole-3,3′-pyrrolizin]-2(1*H*)-one

**DOI:** 10.1107/S2056989015012931

**Published:** 2015-07-11

**Authors:** M. Fathimunnisa, H. Manikandan, S. Selvanayagam, B. Sridhar

**Affiliations:** a Department of Chemistry, Faculty of Science, Annamalai University, Annamalainagar 608 002, India; b Department of Physics, Kings College of Engineering, Punalkulam 613 303, India; c Laboratory of X-ray Crystallography, Indian Institute of Chemical Technology, Hyderabad 500 067, India

**Keywords:** crystal structure, pyrrolizidine derivatives, N—H⋯O hydrogen bonds, C—H⋯π inter­actions

## Abstract

In the title pyrrolizidine derivative, C_33_H_26_F_2_N_2_O_2_, both pyrrolidine rings of the pyrrolizidine moiety adopt an envelope conformation. The di­fluoro­phenyl group is oriented at an angle of 54.3 (1)° with respect to the oxindole moiety. The crystal packing features an N—H⋯O hydrogen bond, which forms an *R*_2_^2^(8) motif, and a C—H⋯O inter­action, which generates a *C*(8) chain along [010]. In addition, this chain structure is stabilized by C—H⋯π inter­actions. In one of the pyrrolidine rings, the methyl­ene group forming the flap of an envelope and the H atoms of the adjacent methyl­ene groups are disordered over two sets of sites, with site-occupancy factors of 0.571 (4) and 0.429 (4)

## Abstract 2

In the title spiro-pyrrolizidine derivative, the di­fluoro­phenyl group is oriented at an angle of 54.3 (1)° with respect to the oxindole moiety. In the crystal, mol­ecules are linked *via* N—H⋯O hydrogen bonds, forming dimers with an 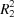
(8) motif.

## Chemical context   

Isatin (1*H*-indole-2,3-dione) has been exploited extensively as a key inter­mediate in organic multicomponent reactions due to its anti­bacterial (Sridhar *et al.*, 2001 ▸), anti­fungal (Amal Raj *et al.*, 2003 ▸; Dandia *et al.*, 2006 ▸), anti­viral (Quenelle *et al.*, 2006 ▸), anti-HIV (Sriram *et al.*, 2006 ▸; Pandeya *et al.*, 2000 ▸), anti-mycobacterial (Feng *et al.*, 2010 ▸), anti­cancer (Gursoy & Karali, 2003 ▸), anti-inflammatory (Sridhar & Ramesh, 2001 ▸) and anti­convulsant (Verma *et al.*, 2004 ▸) activities. The versatile reactivity of isatin has led to the synthesis of a number of isatin-based spiro compounds. Chalcones are precursors and valuable inter­mediates for the synthesis of many biologically important heterocyclic compounds. Therefore, the combination of chalcone with isatin and secondary amino acids provides spiro­oxindolopyrrolizidine derivatives with enhanced biological activities. In view of the many inter­esting applications of pyrrolizidine derivatives, we synthesized the title compound and report herein its crystal structure.
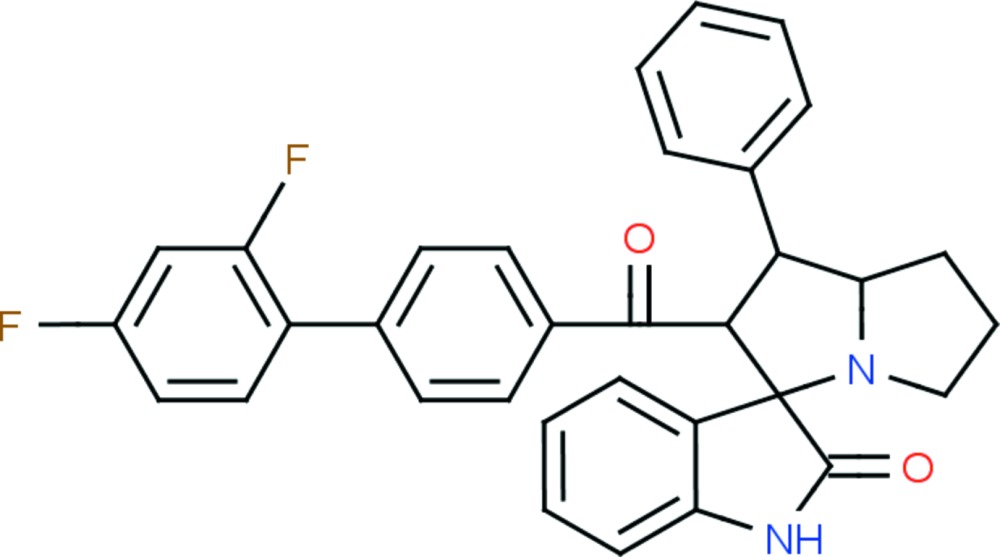


## Structural commentary   

**Figure 1:**
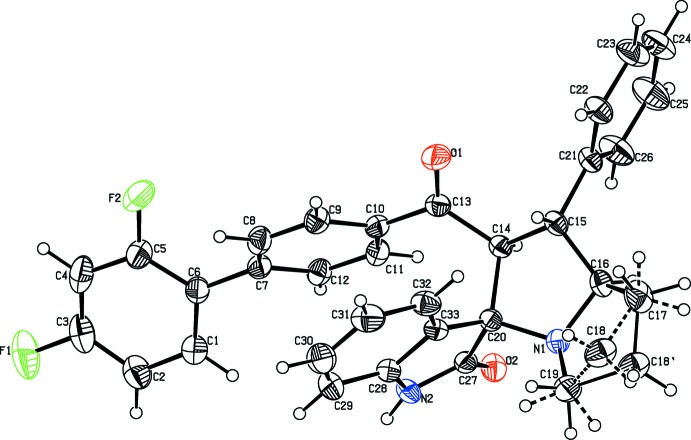
The mol­ecular structure of the title compound, showing the atom labelling. Displacement ellipsoids are drawn at the 30% probability level.

**Figure 2:**
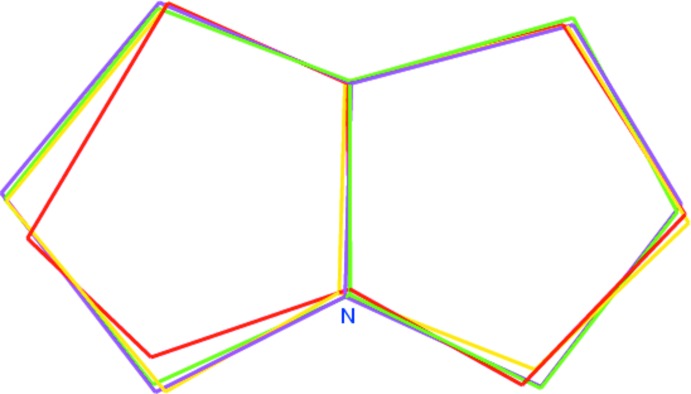
Superposition of pyrrolizidine ring system of (I) (magenta) with the similar reported pyrrolizidine ring system structures in (II) (yellow; Selvanayagam *et al.*, 2010 ▸), (III) (green; Usha *et al.*, 2005 ▸) and (IV) (red; Seshadri *et al.*, 2003 ▸).

The mol­ecular structure of the title compound, (I), is illus­trated in Fig. 1 ▸. The geometry of the pyrrolizidine ring system (N1/C20/C14–C19) in (I) is comparable with that reported for similar structures, namely methyl 4-phenyl-1,2,3,3a,4,4a,5,12c-octa­hydro­naphtho­[1′,2′:3,2]furo[5,4-*b*]pyrrolizine-4a-carboxyl­ate (II) (Selvanayagam *et al.*, 2010 ▸), ethyl 2,2′′-dioxo-2′,3′,5′,6′,7′,7a′-hexa­hydro­acenaphthene-1-spiro-3′-1′*H*-pyrrolizine-2′spiro-1′′-acenaphthene-1-carboxyl­ate (III) (Usha *et al.*, 2005 ▸) and 2′-(*p*-meth­oxy­benzo­yl)-1′,2,2′,3,5′,6′,7′,7a′-octa­hydro-1*H*-indan-2-spiro-3′-(3′*H*- pyrrolizine)-1′-spiro-3′′-1*H*-indoline-1,2′′,3-trione (IV) (Seshadri *et al.*, 2003 ▸). The superposition of the pyrrolizidine ring system of (I) with that in the above-mentioned structures, using *Qmol* (Gans & Shalloway, 2001 ▸), gives an r.m.s. deviation of 0.290 Å between (I) and (II), 0.115 Å between (I) and (III), and 0.389 Å between (I) and (IV); see Fig. 2 ▸.

The sum of the angles at N1 of the pyrrolizidine ring system (340°) is in accordance with *sp* ^3^ hybridization. The fluorine atoms, F1 and F2, deviate by 0.006 (2) and −0.010 (2) Å, respectively, from the plane of the benzene ring (C1–C6) to which they are attached. The oxindole group system is planar with maximum deviations from its plane for the carbonyl C30 [−0.048 (2) Å] and O2 atoms [−0.122 (1) Å]. The di­fluoro­phenyl group is oriented at an angle of 54.3 (1)° with respect to the oxindole moiety. The benzene rings C7–C12 and C21–C26 are oriented at a dihedral angle of 52.7 (1)°. The dihedral angles subtended by these two benzene rings with respect to the oxindole moiety are 21.2 (1) and 31.6 (1)°, respectively. The dihedral angle between the benzene rings of the biphenyl group is 44.3 (1)°. Atom C18 of the pyrrolizidine ring system, and the adjacent methyl­ene group H atoms, are disordered over two sets of sites, with the site-occupancy factors of 0.571 (4) and 0.429 (4).

In the pyrrolizidine ring system, both pyrrolidine rings adopt envelope conformations; the puckering parameters are: *q*_2_ = 0.393 (2) Å and ϕ = −167.8 (2)° for N1/C20/C14–C16 ring, and *q*_2_ = 0.280 (3) Å and ϕ = 104.8 (4)° for N1/C16–C19. In the N1/C20/C14–C16 ring, atom C14 deviates by 0.594 (2) Å from the least-squares plane through the remaining four atoms, whereas in the N1/C16-C19 ring, atoms C18 and C18′ deviate by −0.401 (5) and 0.434 (4) Å, respectively, from the plane through the remaining four atoms.

## Supra­molecular features   

**Figure 3:**
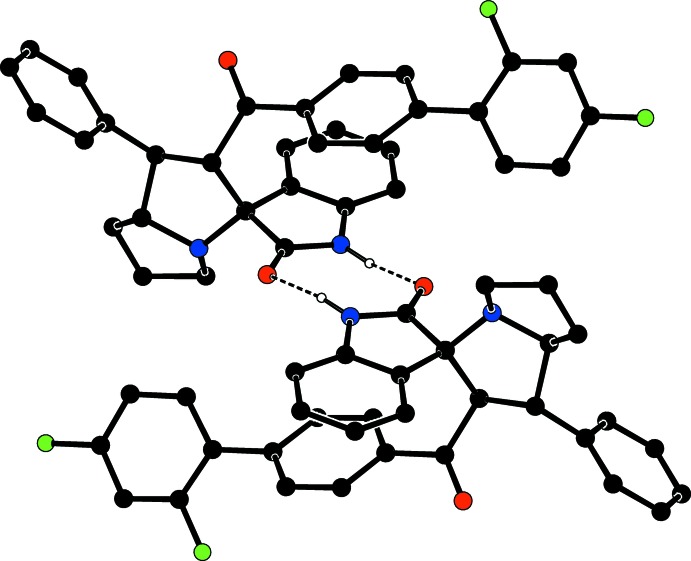
The inversion dimer formed *via* N—H⋯O hydrogen bonds (dashed lines). For clarity H atoms not involved in these hydrogen bonds have been omitted.

**Figure 4:**
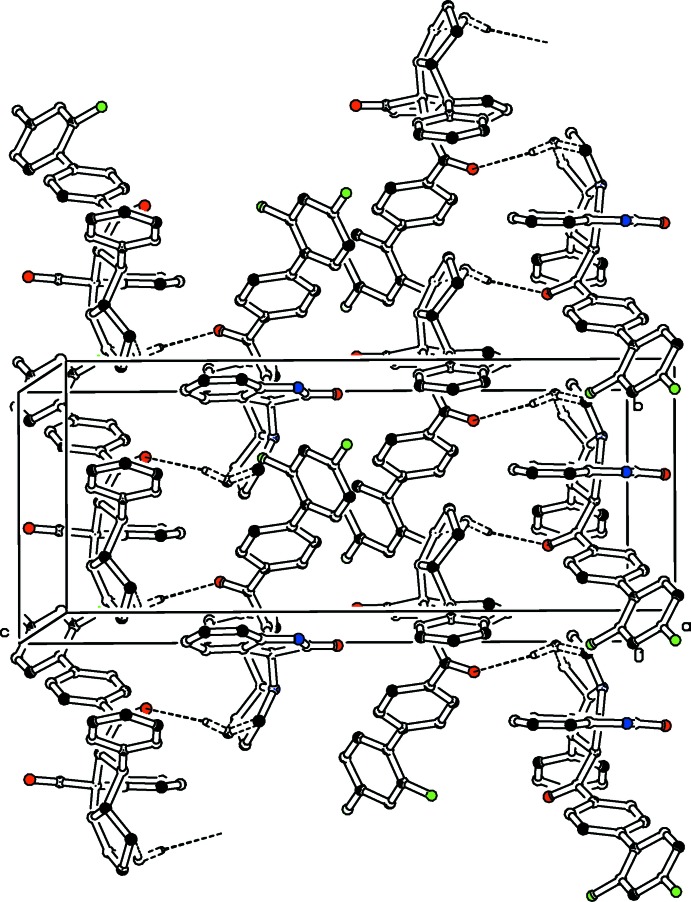
The packing of the title compound, viewed approximately down the *a* axis. C—H⋯O inter­actions are shown as dashed lines (see Table 1 ▸). For clarity, H atoms not involved in these inter­actions have been omitted.

**Figure 5:**
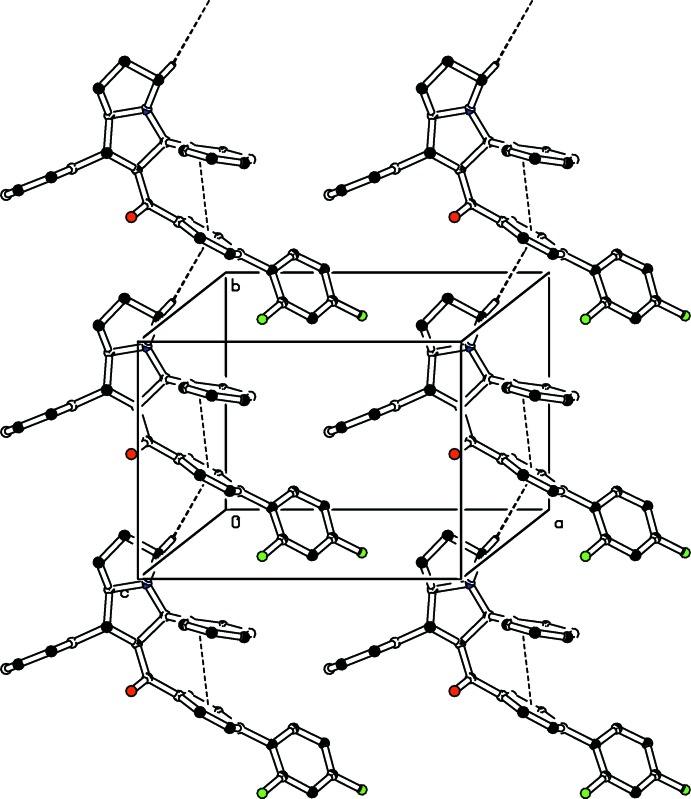
The packing of the title compound, showing the C—H⋯π and π–π inter­actions as dashed lines. For clarity H atoms not involved in these inter­actions have been omitted.

The geometry of inter­actions observed in this structure are given in Table 1 ▸. In the crystal, mol­ecules associate *via* N—H⋯O hydrogen bonds into inversion dimers, generating an 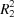
(8) motif; see Fig. 3 ▸. C—H⋯O hydrogen bonds link the mol­ecules, forming *C*(8) chains propagating along [010]; see Fig. 4 ▸. C—H⋯π inter­actions also link the mol­ecules into *C*(8) chains propagating along [010]; see Fig. 5 ▸. In addition, weak intra­molecular π–π inter­actions, involving the benzene ring (C7–C12) and the pyrrolidine ring of the oxindole moiety (C20/C27/N2/C28/C33) stabilize the mol­ecular packing [centroid-to-centroid distance = 3.621 (1) Å].

## Synthesis and crystallization   

To a solution of isatin (1 mmol) and L-proline (1 mmol) in methanol (25 ml), 1-[4-(2,4-di­fluoro­phen­yl)phen­yl]3-phenyl­prop-2-en-1-one (1 mmol) was added and the solution was refluxed for 6–8 h. The completion of reaction was monitored by thin layer chromatography. After completion, the reaction mixture was poured onto crushed ice. The precipitate obtained was filtered and dried at room temperature. Suitable crystals were obtained by slow evaporation of a solution of the title compound in aceto­nitrile at room temperature.

## Refinement   

Crystal data, data collection and structure refinement details are summarized in Table 2 ▸. H atoms were placed in idealized positions and allowed to ride on their parent atoms: C—H = 0.93–0.97 Å, with *U*_iso_(H) = 1.5*U*_eq_(C) for methyl H atoms and 1.2*U*_eq_(C) for other H atoms. Atom C18 is disordered over two positions, with the major component having 0.571 (4) occupancy. Pairs of C—C distances were restrained to 1.54 (1) Å. The temperature factor of C18′ was set to that of C18 with the EADP instruction of *SHELXL2014/7* (Sheldrick, 2015 ▸).

## Supplementary Material

Crystal structure: contains datablock(s) I, global. DOI: 10.1107/S2056989015012931/gk2637sup1.cif

Structure factors: contains datablock(s) I. DOI: 10.1107/S2056989015012931/gk2637Isup2.hkl

Supporting information file. DOI: 10.1107/S2056989015012931/gk2637Isup3.cml

CCDC reference: 1410535

Additional supporting information:  crystallographic information; 3D view; checkCIF report

## Figures and Tables

**Table 1:** Hydrogen-bond geometry (, ) *Cg* is the centroid of the C7C12 ring.

*D*H*A*	*D*H	H*A*	*D* *A*	*D*H*A*
N2H2O2^i^	0.86	2.06	2.854(2)	154
C18H18*B*O1^ii^	0.97	2.36	3.175(6)	141
C19H19*C* *Cg* ^iii^	0.97	2.91	3.659(2)	135

Symmetry codes: (i) 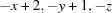; (ii) 
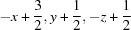
; (iii) 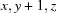.

**Table 2:** Experimental details

Crystal data	
Chemical formula	C_33_H_26_F_2_N_2_O_2_
*M*_r_	520.56
Crystal system, space group	Monoclinic, *P*2_1_/*n*
Temperature (K)	292
*a*, *b*, *c* ()	12.6019(13), 9.3128(10), 22.441(2)
()	98.805(2)
*V* (^3^)	2602.6(5)
*Z*	4
Radiation type	Mo *K*
(mm^1^)	0.09
Crystal size (mm)	0.22 0.20 0.18

Data collection	
Diffractometer	Bruker SMART APEX CCD area detector
No. of measured, independent and observed [*I* > 2(*I*)] reflections	29662, 6300, 4886
*R*_int_	0.025
(sin /)_max_ (^1^)	0.668

Refinement	
*R*[*F* ^2^ > 2(*F* ^2^)], *wR*(*F* ^2^), *S*	0.057, 0.155, 1.04
No. of reflections	6300
No. of parameters	356
No. of restraints	4
H-atom treatment	H-atom parameters constrained
_max_, _min_ (e ^3^)	0.30, 0.20

Computer programs: *SMART* and *SAINT* (Bruker, 2001 ▸), *SHELXS97* (Sheldrick, 2008 ▸), *SHELXL2014* (Sheldrick, 2015 ▸) *ORTEP-3 for Windows* (Farrugia, 2012 ▸) and *PLATON* (Spek, 2009 ▸).

## supplementary crystallographic information

### Crystal data

**Table:**

C_33_H_26_F_2_N_2_O_2_	*D*_x_ = 1.329 Mg m^−^^3^
*M**_r_* = 520.56	Melting point: 451 K
Monoclinic, *P*2_1_/*n*	Mo *K*α radiation, λ = 0.71073 Å
*a* = 12.6019 (13) Å	Cell parameters from 18178 reflections
*b* = 9.3128 (10) Å	θ = 2.2–27.2°
*c* = 22.441 (2) Å	µ = 0.09 mm^−^^1^
β = 98.805 (2)°	*T* = 292 K
*V* = 2602.6 (5) Å^3^	Block, brown
*Z* = 4	0.22 × 0.20 × 0.18 mm
*F*(000) = 1088	

### Data collection

**Table:**

Bruker SMART APEX CCD area-detector diffractometer	*R*_int_ = 0.025
Radiation source: fine-focus sealed tube	θ_max_ = 28.3°, θ_min_ = 1.8°
ω scans	*h* = −16→16
29662 measured reflections	*k* = −12→12
6300 independent reflections	*l* = −29→29
4886 reflections with *I* > 2σ(*I*)	

### Refinement

**Table:**

Refinement on *F*^2^	4 restraints
Least-squares matrix: full	Hydrogen site location: inferred from neighbouring sites
*R*[*F*^2^ > 2σ(*F*^2^)] = 0.057	H-atom parameters constrained
*wR*(*F*^2^) = 0.155	*w* = 1/[σ^2^(*F*_o_^2^) + (0.0756*P*)^2^ + 0.7147*P*] where *P* = (*F*_o_^2^ + 2*F*_c_^2^)/3
*S* = 1.04	(Δ/σ)_max_ < 0.001
6300 reflections	Δρ_max_ = 0.30 e Å^−^^3^
356 parameters	Δρ_min_ = −0.20 e Å^−^^3^

### Special details

**Table:**

Geometry. All esds (except the esd in the dihedral angle between two l.s. planes) are estimated using the full covariance matrix. The cell esds are taken into account individually in the estimation of esds in distances, angles and torsion angles; correlations between esds in cell parameters are only used when they are defined by crystal symmetry. An approximate (isotropic) treatment of cell esds is used for estimating esds involving l.s. planes.

### Fractional atomic coordinates and isotropic or equivalent isotropic displacement parameters (Å^2^)

**Table:**

	*x*	*y*	*z*	*U*_iso_*/*U*_eq_	Occ. (<1)
F1	1.42983 (13)	−0.1726 (2)	0.03334 (8)	0.1156 (6)	
F2	1.15354 (12)	−0.15319 (15)	0.14980 (7)	0.0922 (5)	
O1	0.75919 (11)	0.28958 (15)	0.18945 (6)	0.0609 (4)	
O2	0.86504 (9)	0.55718 (14)	0.00609 (5)	0.0492 (3)	
N1	0.78219 (10)	0.71854 (14)	0.10329 (6)	0.0426 (3)	
N2	1.01135 (10)	0.54887 (15)	0.08094 (6)	0.0457 (3)	
H2	1.0612	0.5404	0.0589	0.055*	
C1	1.21887 (15)	0.0862 (2)	0.03545 (9)	0.0555 (4)	
H1	1.1947	0.1707	0.0158	0.067*	
C2	1.30872 (17)	0.0194 (3)	0.01994 (10)	0.0692 (6)	
H2A	1.3453	0.0587	−0.0092	0.083*	
C3	1.34241 (18)	−0.1056 (3)	0.04851 (11)	0.0738 (6)	
C4	1.29191 (19)	−0.1656 (3)	0.09195 (12)	0.0755 (6)	
H4	1.3160	−0.2509	0.1109	0.091*	
C5	1.20386 (16)	−0.0946 (2)	0.10660 (10)	0.0595 (5)	
C6	1.16360 (13)	0.03136 (17)	0.07927 (8)	0.0463 (4)	
C7	1.06742 (13)	0.10538 (17)	0.09596 (8)	0.0439 (4)	
C8	1.05427 (14)	0.1245 (2)	0.15593 (8)	0.0515 (4)	
H8	1.1059	0.0888	0.1864	0.062*	
C9	0.96640 (14)	0.19527 (19)	0.17080 (8)	0.0495 (4)	
H9	0.9587	0.2058	0.2111	0.059*	
C10	0.88866 (13)	0.25143 (16)	0.12608 (7)	0.0416 (3)	
C11	0.90091 (13)	0.23278 (17)	0.06626 (7)	0.0444 (4)	
H11	0.8496	0.2694	0.0359	0.053*	
C12	0.98902 (13)	0.16000 (17)	0.05137 (8)	0.0450 (4)	
H12	0.9958	0.1475	0.0110	0.054*	
C13	0.79685 (13)	0.33090 (17)	0.14605 (7)	0.0422 (3)	
C14	0.75582 (11)	0.46789 (16)	0.11342 (7)	0.0370 (3)	
H14	0.7254	0.4442	0.0718	0.044*	
C15	0.67061 (12)	0.54637 (17)	0.14317 (7)	0.0396 (3)	
H15	0.6977	0.5545	0.1864	0.048*	
C16	0.67149 (12)	0.69620 (18)	0.11570 (7)	0.0442 (4)	
H16	0.6214	0.6992	0.0777	0.053*	
C17	0.64921 (16)	0.8225 (2)	0.15536 (10)	0.0655 (5)	
H17A	0.6160	0.7899	0.1892	0.079*	0.429 (4)
H17B	0.6022	0.8918	0.1324	0.079*	0.429 (4)
H17C	0.5767	0.8579	0.1433	0.079*	0.571 (4)
H17D	0.6573	0.7934	0.1973	0.079*	0.571 (4)
C18	0.7598 (4)	0.8885 (6)	0.1772 (2)	0.0596 (8)	0.429 (4)
H18A	0.7543	0.9919	0.1808	0.072*	0.429 (4)
H18B	0.7901	0.8494	0.2162	0.072*	0.429 (4)
C18'	0.7266 (3)	0.9325 (4)	0.14717 (19)	0.0596 (8)	0.571 (4)
H18C	0.7450	0.9886	0.1837	0.072*	0.571 (4)
H18D	0.6981	0.9965	0.1145	0.072*	0.571 (4)
C19	0.82659 (15)	0.85131 (19)	0.13188 (10)	0.0574 (5)	
H19A	0.9004	0.8368	0.1506	0.069*	0.429 (4)
H19B	0.8247	0.9274	0.1022	0.069*	0.429 (4)
H19C	0.8629	0.9068	0.1045	0.069*	0.571 (4)
H19D	0.8768	0.8308	0.1681	0.069*	0.571 (4)
C20	0.84415 (12)	0.58513 (16)	0.11279 (7)	0.0364 (3)	
C21	0.56087 (12)	0.47812 (19)	0.13646 (7)	0.0436 (4)	
C22	0.51065 (14)	0.4564 (2)	0.18619 (8)	0.0533 (4)	
H22	0.5462	0.4800	0.2244	0.064*	
C23	0.40755 (16)	0.3997 (2)	0.17992 (11)	0.0683 (6)	
H23	0.3746	0.3860	0.2138	0.082*	
C24	0.35447 (16)	0.3642 (3)	0.12446 (11)	0.0729 (6)	
H24	0.2856	0.3259	0.1204	0.088*	
C25	0.40302 (17)	0.3850 (3)	0.07466 (11)	0.0801 (7)	
H25	0.3667	0.3616	0.0366	0.096*	
C26	0.50557 (16)	0.4405 (3)	0.08058 (9)	0.0691 (6)	
H26	0.5381	0.4528	0.0464	0.083*	
C27	0.90604 (12)	0.56227 (16)	0.05899 (7)	0.0388 (3)	
C28	1.02933 (13)	0.55045 (17)	0.14422 (8)	0.0441 (4)	
C29	1.12487 (15)	0.5294 (2)	0.18212 (10)	0.0608 (5)	
H29	1.1885	0.5134	0.1670	0.073*	
C30	1.12263 (17)	0.5329 (3)	0.24354 (10)	0.0714 (6)	
H30	1.1858	0.5176	0.2701	0.086*	
C31	1.02894 (18)	0.5586 (2)	0.26619 (9)	0.0666 (6)	
H31	1.0297	0.5612	0.3077	0.080*	
C32	0.93354 (15)	0.5804 (2)	0.22761 (8)	0.0520 (4)	
H32	0.8702	0.5983	0.2429	0.062*	
C33	0.93381 (12)	0.57534 (16)	0.16614 (7)	0.0399 (3)	

### Atomic displacement parameters (Å^2^)

**Table:**

	*U*^11^	*U*^22^	*U*^33^	*U*^12^	*U*^13^	*U*^23^
F1	0.0836 (10)	0.1401 (15)	0.1255 (13)	0.0590 (10)	0.0234 (9)	−0.0066 (11)
F2	0.1023 (10)	0.0606 (8)	0.1199 (12)	0.0165 (7)	0.0365 (9)	0.0358 (7)
O1	0.0647 (8)	0.0607 (8)	0.0624 (8)	0.0027 (6)	0.0259 (6)	0.0170 (6)
O2	0.0497 (7)	0.0612 (7)	0.0390 (6)	0.0094 (5)	0.0141 (5)	0.0031 (5)
N1	0.0406 (7)	0.0391 (7)	0.0500 (7)	0.0036 (5)	0.0127 (6)	−0.0004 (6)
N2	0.0352 (7)	0.0518 (8)	0.0534 (8)	0.0014 (6)	0.0169 (6)	0.0000 (6)
C1	0.0533 (10)	0.0548 (10)	0.0576 (11)	0.0060 (8)	0.0059 (8)	0.0008 (8)
C2	0.0567 (12)	0.0838 (15)	0.0681 (13)	0.0105 (11)	0.0127 (10)	−0.0012 (11)
C3	0.0564 (12)	0.0861 (16)	0.0771 (15)	0.0250 (11)	0.0040 (11)	−0.0130 (12)
C4	0.0725 (14)	0.0603 (13)	0.0888 (16)	0.0262 (11)	−0.0034 (12)	0.0028 (11)
C5	0.0590 (11)	0.0476 (10)	0.0704 (12)	0.0051 (9)	0.0048 (9)	0.0062 (9)
C6	0.0427 (8)	0.0398 (8)	0.0538 (9)	0.0012 (7)	−0.0011 (7)	−0.0036 (7)
C7	0.0421 (8)	0.0341 (8)	0.0542 (9)	−0.0016 (6)	0.0035 (7)	−0.0007 (7)
C8	0.0502 (9)	0.0526 (10)	0.0488 (9)	0.0065 (8)	−0.0017 (7)	0.0067 (8)
C9	0.0538 (10)	0.0495 (9)	0.0444 (9)	0.0028 (8)	0.0054 (7)	0.0051 (7)
C10	0.0428 (8)	0.0335 (7)	0.0479 (9)	−0.0024 (6)	0.0055 (7)	0.0022 (6)
C11	0.0464 (9)	0.0381 (8)	0.0457 (9)	0.0027 (7)	−0.0022 (7)	−0.0012 (7)
C12	0.0501 (9)	0.0391 (8)	0.0445 (9)	0.0023 (7)	0.0027 (7)	−0.0037 (7)
C13	0.0411 (8)	0.0418 (8)	0.0437 (8)	−0.0059 (6)	0.0065 (6)	0.0008 (7)
C14	0.0335 (7)	0.0415 (8)	0.0369 (7)	−0.0009 (6)	0.0083 (6)	−0.0002 (6)
C15	0.0329 (7)	0.0515 (9)	0.0352 (7)	0.0002 (6)	0.0075 (6)	−0.0035 (6)
C16	0.0377 (8)	0.0491 (9)	0.0464 (9)	0.0063 (7)	0.0086 (6)	−0.0045 (7)
C17	0.0613 (12)	0.0610 (12)	0.0785 (14)	0.0111 (9)	0.0248 (10)	−0.0178 (10)
C18	0.081 (2)	0.0434 (17)	0.059 (2)	0.0007 (14)	0.0238 (18)	−0.0071 (13)
C18'	0.081 (2)	0.0434 (17)	0.059 (2)	0.0007 (14)	0.0238 (18)	−0.0071 (13)
C19	0.0568 (10)	0.0402 (9)	0.0761 (13)	−0.0026 (8)	0.0127 (9)	−0.0041 (8)
C20	0.0360 (7)	0.0383 (8)	0.0361 (7)	0.0008 (6)	0.0100 (6)	0.0007 (6)
C21	0.0337 (7)	0.0528 (9)	0.0453 (8)	0.0018 (7)	0.0096 (6)	−0.0011 (7)
C22	0.0433 (9)	0.0679 (12)	0.0514 (10)	−0.0011 (8)	0.0159 (7)	−0.0054 (8)
C23	0.0537 (11)	0.0800 (14)	0.0789 (14)	−0.0079 (10)	0.0350 (10)	−0.0065 (11)
C24	0.0433 (10)	0.0815 (15)	0.0966 (17)	−0.0167 (10)	0.0188 (11)	−0.0200 (13)
C25	0.0536 (12)	0.114 (2)	0.0702 (14)	−0.0231 (13)	0.0027 (10)	−0.0201 (13)
C26	0.0494 (10)	0.1077 (18)	0.0503 (11)	−0.0186 (11)	0.0077 (8)	−0.0067 (11)
C27	0.0398 (8)	0.0361 (7)	0.0433 (8)	0.0023 (6)	0.0151 (6)	0.0034 (6)
C28	0.0390 (8)	0.0403 (8)	0.0528 (9)	−0.0031 (6)	0.0062 (7)	0.0032 (7)
C29	0.0388 (9)	0.0633 (12)	0.0782 (13)	0.0002 (8)	0.0025 (9)	0.0065 (10)
C30	0.0536 (11)	0.0810 (15)	0.0715 (14)	−0.0087 (10)	−0.0167 (10)	0.0174 (11)
C31	0.0689 (13)	0.0795 (14)	0.0469 (10)	−0.0198 (11)	−0.0051 (9)	0.0107 (9)
C32	0.0521 (10)	0.0615 (11)	0.0420 (9)	−0.0122 (8)	0.0061 (7)	0.0022 (8)
C33	0.0369 (7)	0.0389 (8)	0.0441 (8)	−0.0050 (6)	0.0069 (6)	0.0019 (6)

### Geometric parameters (Å, º)

**Table:**

F1—C3	1.354 (2)	C17—C18'	1.446 (4)
F2—C5	1.352 (2)	C17—C18	1.534 (5)
O1—C13	1.2096 (19)	C17—H17A	0.9700
O2—C27	1.2211 (19)	C17—H17B	0.9700
N1—C19	1.464 (2)	C17—H17C	0.9700
N1—C20	1.4655 (19)	C17—H17D	0.9700
N1—C16	1.478 (2)	C18—C19	1.457 (5)
N2—C27	1.349 (2)	C18—H18A	0.9700
N2—C28	1.403 (2)	C18—H18B	0.9700
N2—H2	0.8600	C18'—C19	1.552 (4)
C1—C2	1.382 (3)	C18'—H18C	0.9700
C1—C6	1.387 (3)	C18'—H18D	0.9700
C1—H1	0.9300	C19—H19A	0.9700
C2—C3	1.365 (3)	C19—H19B	0.9700
C2—H2A	0.9300	C19—H19C	0.9700
C3—C4	1.362 (3)	C19—H19D	0.9700
C4—C5	1.374 (3)	C20—C33	1.518 (2)
C4—H4	0.9300	C20—C27	1.549 (2)
C5—C6	1.383 (2)	C21—C22	1.380 (2)
C6—C7	1.491 (2)	C21—C26	1.383 (3)
C7—C12	1.391 (2)	C22—C23	1.390 (3)
C7—C8	1.392 (2)	C22—H22	0.9300
C8—C9	1.373 (2)	C23—C24	1.361 (3)
C8—H8	0.9300	C23—H23	0.9300
C9—C10	1.393 (2)	C24—C25	1.367 (3)
C9—H9	0.9300	C24—H24	0.9300
C10—C11	1.385 (2)	C25—C26	1.379 (3)
C10—C13	1.499 (2)	C25—H25	0.9300
C11—C12	1.385 (2)	C26—H26	0.9300
C11—H11	0.9300	C28—C29	1.378 (2)
C12—H12	0.9300	C28—C33	1.388 (2)
C13—C14	1.522 (2)	C29—C30	1.383 (3)
C14—C15	1.534 (2)	C29—H29	0.9300
C14—C20	1.561 (2)	C30—C31	1.376 (3)
C14—H14	0.9800	C30—H30	0.9300
C15—C21	1.508 (2)	C31—C32	1.385 (3)
C15—C16	1.526 (2)	C31—H31	0.9300
C15—H15	0.9800	C32—C33	1.381 (2)
C16—C17	1.527 (2)	C32—H32	0.9300
C16—H16	0.9800		
			
C19—N1—C20	119.52 (13)	C18'—C17—H17D	110.4
C19—N1—C16	110.16 (13)	C16—C17—H17D	110.4
C20—N1—C16	110.51 (12)	H17C—C17—H17D	108.6
C27—N2—C28	111.48 (13)	C19—C18—C17	106.0 (3)
C27—N2—H2	124.3	C19—C18—H18A	110.5
C28—N2—H2	124.3	C17—C18—H18A	110.5
C2—C1—C6	122.21 (19)	C19—C18—H18B	110.5
C2—C1—H1	118.9	C17—C18—H18B	110.5
C6—C1—H1	118.9	H18A—C18—H18B	108.7
C3—C2—C1	118.2 (2)	C17—C18'—C19	105.6 (2)
C3—C2—H2A	120.9	C17—C18'—H18C	110.6
C1—C2—H2A	120.9	C19—C18'—H18C	110.6
F1—C3—C4	118.6 (2)	C17—C18'—H18D	110.6
F1—C3—C2	118.8 (2)	C19—C18'—H18D	110.6
C4—C3—C2	122.7 (2)	H18C—C18'—H18D	108.8
C3—C4—C5	117.2 (2)	C18—C19—N1	106.5 (2)
C3—C4—H4	121.4	N1—C19—C18'	103.87 (18)
C5—C4—H4	121.4	C18—C19—H19A	110.4
F2—C5—C4	117.51 (18)	N1—C19—H19A	110.4
F2—C5—C6	118.67 (18)	C18—C19—H19B	110.4
C4—C5—C6	123.8 (2)	N1—C19—H19B	110.4
C5—C6—C1	115.88 (17)	H19A—C19—H19B	108.6
C5—C6—C7	122.52 (17)	N1—C19—H19C	111.0
C1—C6—C7	121.59 (15)	C18'—C19—H19C	111.0
C12—C7—C8	118.18 (16)	N1—C19—H19D	111.0
C12—C7—C6	120.24 (16)	C18'—C19—H19D	111.0
C8—C7—C6	121.57 (15)	H19C—C19—H19D	109.0
C9—C8—C7	121.07 (16)	N1—C20—C33	118.78 (13)
C9—C8—H8	119.5	N1—C20—C27	108.89 (12)
C7—C8—H8	119.5	C33—C20—C27	101.75 (12)
C8—C9—C10	120.65 (16)	N1—C20—C14	103.27 (11)
C8—C9—H9	119.7	C33—C20—C14	113.37 (12)
C10—C9—H9	119.7	C27—C20—C14	110.84 (12)
C11—C10—C9	118.73 (15)	C22—C21—C26	117.80 (16)
C11—C10—C13	123.87 (14)	C22—C21—C15	120.53 (15)
C9—C10—C13	117.40 (15)	C26—C21—C15	121.62 (15)
C12—C11—C10	120.51 (15)	C21—C22—C23	120.75 (18)
C12—C11—H11	119.7	C21—C22—H22	119.6
C10—C11—H11	119.7	C23—C22—H22	119.6
C11—C12—C7	120.86 (16)	C24—C23—C22	120.42 (19)
C11—C12—H12	119.6	C24—C23—H23	119.8
C7—C12—H12	119.6	C22—C23—H23	119.8
O1—C13—C10	119.98 (15)	C23—C24—C25	119.61 (19)
O1—C13—C14	120.52 (15)	C23—C24—H24	120.2
C10—C13—C14	119.42 (13)	C25—C24—H24	120.2
C13—C14—C15	113.52 (13)	C24—C25—C26	120.3 (2)
C13—C14—C20	113.61 (12)	C24—C25—H25	119.9
C15—C14—C20	102.61 (12)	C26—C25—H25	119.9
C13—C14—H14	108.9	C25—C26—C21	121.12 (19)
C15—C14—H14	108.9	C25—C26—H26	119.4
C20—C14—H14	108.9	C21—C26—H26	119.4
C21—C15—C16	114.09 (13)	O2—C27—N2	126.71 (14)
C21—C15—C14	116.62 (13)	O2—C27—C20	125.07 (14)
C16—C15—C14	102.18 (12)	N2—C27—C20	108.22 (13)
C21—C15—H15	107.8	C29—C28—C33	121.90 (17)
C16—C15—H15	107.8	C29—C28—N2	127.86 (16)
C14—C15—H15	107.8	C33—C28—N2	110.23 (14)
N1—C16—C15	105.67 (12)	C28—C29—C30	117.55 (19)
N1—C16—C17	105.33 (14)	C28—C29—H29	121.2
C15—C16—C17	117.11 (15)	C30—C29—H29	121.2
N1—C16—H16	109.5	C31—C30—C29	121.45 (18)
C15—C16—H16	109.5	C31—C30—H30	119.3
C17—C16—H16	109.5	C29—C30—H30	119.3
C18'—C17—C16	106.51 (19)	C30—C31—C32	120.43 (19)
C16—C17—C18	104.8 (2)	C30—C31—H31	119.8
C16—C17—H17A	110.8	C32—C31—H31	119.8
C18—C17—H17A	110.8	C33—C32—C31	119.01 (18)
C16—C17—H17B	110.8	C33—C32—H32	120.5
C18—C17—H17B	110.8	C31—C32—H32	120.5
H17A—C17—H17B	108.9	C32—C33—C28	119.65 (15)
C18'—C17—H17C	110.4	C32—C33—C20	132.10 (15)
C16—C17—H17C	110.4	C28—C33—C20	108.17 (13)
			
C6—C1—C2—C3	0.9 (3)	C16—N1—C19—C18	17.6 (3)
C1—C2—C3—F1	179.5 (2)	C20—N1—C19—C18'	−145.4 (2)
C1—C2—C3—C4	−0.9 (4)	C16—N1—C19—C18'	−15.8 (2)
F1—C3—C4—C5	179.6 (2)	C17—C18'—C19—N1	27.8 (3)
C2—C3—C4—C5	−0.1 (4)	C19—N1—C20—C33	19.1 (2)
C3—C4—C5—F2	−179.8 (2)	C16—N1—C20—C33	−110.25 (15)
C3—C4—C5—C6	1.0 (3)	C19—N1—C20—C27	−96.55 (17)
F2—C5—C6—C1	179.88 (18)	C16—N1—C20—C27	134.06 (13)
C4—C5—C6—C1	−1.0 (3)	C19—N1—C20—C14	145.62 (14)
F2—C5—C6—C7	0.5 (3)	C16—N1—C20—C14	16.22 (15)
C4—C5—C6—C7	179.63 (19)	C13—C14—C20—N1	−157.37 (12)
C2—C1—C6—C5	0.0 (3)	C15—C14—C20—N1	−34.41 (14)
C2—C1—C6—C7	179.36 (18)	C13—C14—C20—C33	−27.52 (17)
C5—C6—C7—C12	−136.73 (18)	C15—C14—C20—C33	95.44 (14)
C1—C6—C7—C12	43.9 (2)	C13—C14—C20—C27	86.17 (15)
C5—C6—C7—C8	44.6 (2)	C15—C14—C20—C27	−150.87 (12)
C1—C6—C7—C8	−134.78 (19)	C16—C15—C21—C22	−111.32 (18)
C12—C7—C8—C9	0.0 (3)	C14—C15—C21—C22	129.83 (17)
C6—C7—C8—C9	178.73 (16)	C16—C15—C21—C26	66.1 (2)
C7—C8—C9—C10	−0.8 (3)	C14—C15—C21—C26	−52.7 (2)
C8—C9—C10—C11	0.9 (3)	C26—C21—C22—C23	−0.5 (3)
C8—C9—C10—C13	−178.25 (16)	C15—C21—C22—C23	177.01 (18)
C9—C10—C11—C12	−0.2 (2)	C21—C22—C23—C24	0.2 (3)
C13—C10—C11—C12	178.90 (15)	C22—C23—C24—C25	−0.2 (4)
C10—C11—C12—C7	−0.6 (2)	C23—C24—C25—C26	0.6 (4)
C8—C7—C12—C11	0.7 (2)	C24—C25—C26—C21	−1.0 (4)
C6—C7—C12—C11	−178.04 (15)	C22—C21—C26—C25	0.9 (3)
C11—C10—C13—O1	143.39 (17)	C15—C21—C26—C25	−176.6 (2)
C9—C10—C13—O1	−37.5 (2)	C28—N2—C27—O2	−175.97 (15)
C11—C10—C13—C14	−39.9 (2)	C28—N2—C27—C20	3.84 (17)
C9—C10—C13—C14	139.20 (15)	N1—C20—C27—O2	−56.15 (19)
O1—C13—C14—C15	3.2 (2)	C33—C20—C27—O2	177.63 (15)
C10—C13—C14—C15	−173.45 (13)	C14—C20—C27—O2	56.79 (19)
O1—C13—C14—C20	119.99 (17)	N1—C20—C27—N2	124.03 (13)
C10—C13—C14—C20	−56.69 (18)	C33—C20—C27—N2	−2.19 (16)
C13—C14—C15—C21	−72.60 (17)	C14—C20—C27—N2	−123.03 (13)
C20—C14—C15—C21	164.38 (13)	C27—N2—C28—C29	175.07 (17)
C13—C14—C15—C16	162.29 (12)	C27—N2—C28—C33	−4.09 (19)
C20—C14—C15—C16	39.26 (14)	C33—C28—C29—C30	0.4 (3)
C19—N1—C16—C15	−125.74 (14)	N2—C28—C29—C30	−178.66 (18)
C20—N1—C16—C15	8.51 (16)	C28—C29—C30—C31	−0.9 (3)
C19—N1—C16—C17	−1.15 (19)	C29—C30—C31—C32	0.6 (3)
C20—N1—C16—C17	133.09 (15)	C30—C31—C32—C33	0.3 (3)
C21—C15—C16—N1	−156.70 (13)	C31—C32—C33—C28	−0.9 (3)
C14—C15—C16—N1	−29.93 (15)	C31—C32—C33—C20	175.64 (17)
C21—C15—C16—C17	86.42 (18)	C29—C28—C33—C32	0.5 (3)
C14—C15—C16—C17	−146.81 (15)	N2—C28—C33—C32	179.70 (15)
N1—C16—C17—C18'	19.2 (3)	C29—C28—C33—C20	−176.78 (16)
C15—C16—C17—C18'	136.3 (2)	N2—C28—C33—C20	2.44 (18)
N1—C16—C17—C18	−14.8 (3)	N1—C20—C33—C32	63.6 (2)
C15—C16—C17—C18	102.3 (3)	C27—C20—C33—C32	−176.98 (17)
C16—C17—C18—C19	25.7 (4)	C14—C20—C33—C32	−57.9 (2)
C16—C17—C18'—C19	−28.9 (3)	N1—C20—C33—C28	−119.62 (15)
C17—C18—C19—N1	−26.6 (4)	C27—C20—C33—C28	−0.18 (16)
C20—N1—C19—C18	−111.9 (3)	C14—C20—C33—C28	118.87 (14)

### Hydrogen-bond geometry (Å, º)

Cg is the centroid of the C7–C12 ring.

**Table:**

*D*—H···*A*	*D*—H	H···*A*	*D*···*A*	*D*—H···*A*
N2—H2···O2^i^	0.86	2.06	2.854 (2)	154
C18—H18*B*···O1^ii^	0.97	2.36	3.175 (6)	141
C19—H19*C*···*Cg*^iii^	0.97	2.91	3.659 (2)	135

Symmetry codes: (i) −*x*+2, −*y*+1, −*z*; (ii) −*x*+3/2, *y*+1/2, −*z*+1/2; (iii) *x*, *y*+1, *z*.
